# An Interference Mitigation Method for FMCW Radar Based on Time–Frequency Distribution and Dual-Domain Fusion Filtering

**DOI:** 10.3390/s24113288

**Published:** 2024-05-21

**Authors:** Yu Zhou, Ronggang Cao, Anqi Zhang, Ping Li

**Affiliations:** 1 School of Electrical and Mechanical, Beijing Institute of Technology, Beijing 100081, China; 2 Science and Technology on Electromechanical Dynamic Control Laboratory, Beijing Institute of Technology, Beijing 100081, China; 3 Tangshan Research Institute, Beijing Institute of Technology, Tangshan 063611, China

**Keywords:** interference mitigation, time–frequency transform, synchrosqueezed transform, dual-tree complex wavelet transform, convolutional neural network

## Abstract

Radio frequency interference (RFI) significantly hampers the target detection performance of frequency-modulated continuous-wave radar. To address the problem and maintain the target echo signal, this paper proposes a priori assumption on the interference component nature in the radar received signal, as well as a method for interference estimation and mitigation via time–frequency analysis. The solution employs Fourier synchrosqueezed transform to implement the radar’s beat signal transformation from time domain to time–frequency domain, thus converting the interference mitigation to the task of time–frequency distribution image restoration. The solution proposes the use of image processing based on the dual-tree complex wavelet transform and combines it with the spatial domain-based approach, thereby establishing a dual-domain fusion interference filter for time–frequency distribution images. This paper also presents a convolutional neural network model of structurally improved UNet++, which serves as the interference estimator. The proposed solution demonstrated its capability against various forms of RFI through the simulation experiment and showed a superior interference mitigation performance over other CNN model-based approaches.

## 1. Introduction

Frequency-modulated continuous wave (FMCW) radar emits continuous wave signals through a linearly-modulated transmitter and measures the distance and velocity of targets using the frequency difference between the received signal and the transmitted signal. The FMCW radar is widely used in both military and civilian fields. In military applications, FMCW radar is widely used in target reconnaissance and tracking, battlefield surveillance, air defense, missile defense, and ship navigation [1,2,3,4]. These applications rely on the high resolution and low probability of intercept characteristics of FMCW radar. The civilian applications of FMCW radar include meteorological observation, air traffic control, ocean monitoring, ground remote sensing, and intelligent driving [5,6,7,8,9]. In recent years, FMCW radar has been widely adopted in the emerging field of autonomous driving technology, due to its robust detection performance and good accuracy [10,11,12,13].

In practical applications, FMCW radars are confronted with a multitude of active interferences. The interferences can be categorized into coherent and non-coherent interference, in terms of the coherence between the interference signal and the radar signal. Coherent interference, represented by interrupted-sampling repeater jamming (ISRJ), can intercept and retransmit radar signals, accurately simulating target response to achieve the deception jamming effect of false targets. This type of interference is characterized by high synchronization, strong specificity, and a high jamming success rate, but the implementation principles and technologies are complex. Non-coherent interference is a more common type, with a wide distribution of radiation sources and a variety of subtypes, which severely affect the target detection capability of FMCW radars as well [14,15]. Typical non-coherent interferences radiated by jammers in the battlefield environment include noise blanket jamming and some kinds of periodic modulation interference [16,17,18]. Noise blanket jamming can be implemented in the form of broadband, narrowband, or pulse noise with short-term high-power characteristics. This jamming type causes the radar receiver to saturate within its dynamic range, thereby reducing the radar’s target detection ability [19,20,21,22]. Periodic modulation interference includes active jamming from the jammer and mutual interference from other radars. It can also achieve a close approximation, or even overlap with the radar signal spectrum. For a certain radar, the mutual interference from other radars can be considered as a jamming signal with a frequency that varies over time, which may result in false targets with dynamic characteristics, making it difficult for the radar to distinguish them from the real target echoes. The periodic modulation interference signals can be very close, or even overlap with, the working spectrum of FMCW radar, causing false alarms on the radar. These interference signals usually exhibit non-stationarity, with their frequency components, amplitudes, and phases being time-varying. In complex situations, even modulation methods and modulation parameters may dynamically change, making it difficult for traditional methods of frequency domain filtering to effectively remove interference signals. It should be noted that we previously mentioned both jamming and interference; jamming generally refers to the intentional actions of a jammer, while interference is unintentional. This paper focuses on the mitigation of non-coherent interference, which includes both intentional jamming and unintentional interference. To ensure consistency and conciseness in the subsequent descriptions, we will use the term “interference” to collectively refer to both intentional jamming and unintentional interference.

In the field of radar interference mitigation, researchers are dedicated to developing innovative signal processing techniques. These approaches, which employ sophisticated signal reconstruction and adaptive filtering strategies, significantly enhance radar systems’ capabilities to detect and suppress interference in complex environments. Ref. [23] introduces an interference mitigation method that utilizes particle swarm optimization and adaptive filtering, minimizes waveform correlation to mitigate false alarms, and exploits orthogonal receiver properties for interference cancellation, thereby bolstering the radar’s resilience against interference. Ref. [24] presents an RFI mitigation approach based on forward continuous mean excision (FCME) of the instantaneous spectrum. This approach employs kurtosis-based statistical tests for echo signal detection in the time–frequency domain and employs FCME to thoroughly eliminate RFI, while preserving useful target signals. Ref. [25] proposes a matrix pencil (MP)-based method to suppress interference by reconstructing contaminated beat frequency signal samples. Ref. [26] employs constant false alarm rate (CFAR) detectors to detect interference spectra in the time–frequency domain, thereby achieving interference suppression. These methods, by refining signal processing strategies, effectively enhance radar performance in interference-laden environments. Ref. [27] details an FMCW radar interference mitigation technique based on short-time Fourier transform (STFT) domain interpolation, phase matching, and reconstructive linear prediction. This technique is non-iterative, efficient, and FFT-compatible. Ref. [28] advocates for interference mitigation in the correlation domain, using matched filtering combined with autoregressive models. Ref. [29] employs variational mode decomposition to separate interference from target signals, obtaining interference-free targets through signal reconstruction. Ref. [30] presents an enhanced two-dimensional notch filter based on image segmentation for RFI mitigation with signal protection capability. Ref. [31] proposes a rapid interference mitigation method based on signal spectrum sub-bands, involving interference mitigation for each sub-band separately, and signal reconstruction. Ref. [32] proposes an interference detection and mitigation method for FMCW radar systems based on time-domain signal reconstruction. This method identifies interference through differences between targets and interference in one-dimensional fast Fourier transform (1D-FFT) results, then removes interference in the time domain to mitigate its impact on target signals. Despite their effectiveness, these methods are often tailored to specific scenarios, limiting their applicability to broader interference mitigation contexts.

In addition to traditional signal processing methods, the advent of sparse signal representation and sparse reconstruction techniques has opened new avenues for FMCW radar interference mitigation. The sparsity of signals allows for the efficient representation of signals using fewer coefficients, which is particularly useful in scenarios where the radar system is subjected to non-stationary interference. Recent advances in this area have led to the development of algorithms that can accurately identify and mitigate interference, thereby enhancing the radar’s ability to operate effectively in challenging environments. In Ref. [33], a method based on the proximal gradient descent was proposed, exploiting the temporal sparsity of cross-interference and the sparsity of interference-free baseband signals in the range-Doppler frequency domain. This approach aims to improve the estimation and elimination of cross-interference, thus enhancing the radar’s resilience against interference. Furthermore, Ref. [34] proposed a method to detect the interference envelope using a low-pass filter and treated the interference area as missing data, restoring the radar echo using the least squares method with L1 norm regularization. This method demonstrates the potential of sparse reconstruction techniques in mitigating interference and preserving the integrity of radar signals. Ref. [35] proposed a unified tensor-based algorithm for parameter estimation of targets and channels. This method deals with tensor decomposition using sparsity-promoting priors, allowing the algorithm to effectively recover channel state and target parameters from observational data, without prior knowledge of the target. Ref. [36] suggested using the sparsity of atomic norm and L1 norm of the signal for estimating the target delays and Doppler shifts, thereby obtaining the target’s position and motion status. In Ref. [37], the employment of the fast orthogonal matching pursuit (OMP) algorithm facilitated a process wherein the interfering signal was mapped onto a truncated chirp-let basis. This approach achieved the segregation of the target signal from the interference, with negligible information loss. Ref. [38] formulated an optimization challenge, which involves the analysis of the target echo sparsity and the row sparsity of the useful echo signals within the frequency spectrum. By imposing row sparsity constraints, the target echo can be accurately extracted.

The rapid development of deep learning technology has led to the emergence of radar interference mitigation methods based on neural networks, which have demonstrated considerable potential and advantages. These methods, which learn the complex patterns and features of the data, provide a more flexible and robust means of interference mitigation, significantly improving the adaptability and accuracy of radar systems when faced with complex interference scenarios. In Ref. [39], a deep learning method based on contrastive learning was proposed, which enhances the receptive field of the neural network through dilated convolution and trains it with a specific contrastive learning strategy to better distinguish between interference and desired signals. In Ref. [40], the interference mitigation problem was treated as a regression issue and a method for interference mitigation, based on complex-valued convolutional neural networks, was proposed, which processes the time–frequency distribution of radar beat signals to achieve interference suppression. In Ref. [41], recurrent neural network (RNN) and self-attention mechanisms were employed to enhance the performance of interference suppression. These studies indicate that deep learning methods exhibit greater flexibility and robustness in addressing complex interference scenarios. Inspired by approximate message passing, Ref. [42] designed a feature-guided unsupervised adaptive neural network, transforming the interference mitigation task into an optimization task with a regularization term. In Ref. [43], the researchers proposed the use of generative adversarial networks (GANs) for interference mitigation. This approach involves extracting target echo features through the discriminator network and generating a time–frequency distribution image of interference-free signals through the generator network.

Driven by development of deep learning, radar interference mitigation methods based on neural networks have demonstrated excellent performance in dealing with complex interference scenarios. By learning the complex patterns and features of the data, these methods provide more flexible and robust processing means for interference. To further explore this field, this paper proposes an interference mitigation method that cooperates time–frequency transform with deep neural network algorithms, extending their application to the mitigation of more non-coherent interference types. The paper first proposes an a priori assumption about the interference components in the time–frequency distribution and defines the interference mitigation model based on this. Secondly, it takes the use of Fourier synchrosqueezed transform (FSST) to convert the beat signal of the radar from the time domain to the time–frequency domain. This is performed in order to utilize the advantages of high time–frequency resolution and high energy aggregation, which help to accurately distinguish the energy distribution differences between the target echo components and the interference components. On this basis, a dual-domain fusion approach is proposed for the processing of the beat signal time–frequency distribution. This involves the design of an improved model of the convolutional neural network UNet++ for the estimation and mitigation of interference components. The dual-domain fusion interference mitigation result is employed to obtain the time-domain form of an interference-free beat signal, through the inverse transform of FSST. This approach enables the interference to be mitigated, while accurately preserving and enhancing the energy intensity of the target echo components, thereby protecting the normal progress of subsequent work of FMCW radar.

The rest of the paper is organized as follows. Section 2 establishes the model of the problem concerned. Section 3 details the interference mitigation method. Section 4 introduces the simulation experiment setup and performance evaluation method. Section 5 provides the analysis of the experimental results. Section 6 discusses the innovations and the essence of the proposed method. Section 7 summarizes the whole paper.

## 2. Modeling of Signal and Task

**Figure 1:**
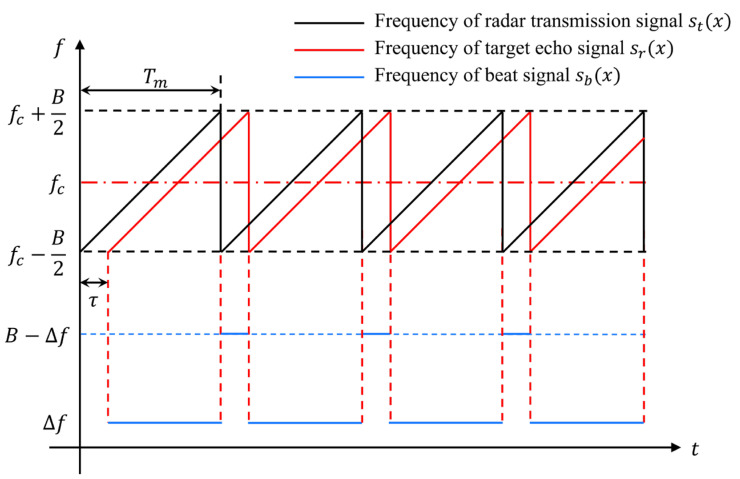
Principle of LFM signal of FMCW radar.

The principle of target range detection by FMCW radar through the frequency relationship of linear frequency modulated (LFM) signal is shown in Figure 1. $f_{c}$ denotes the center frequency; B denotes the modulation bandwidth; $T_{m}$ denotes the modulation period, and Δf denotes the beat frequency. Considering a single pulse period, the transmission signal $s_{t}\left(t\right)$ can be expressed as Equation (1).

(1) $$s_{t}\left(t\right)=a_{0}exp\left[j2\pi \left(f_{c}t+\frac{1}{2}kt^{2}\right)\right]$$

where the chirp rate is $k=B/T_{m}$. The signal $s_{t}\left(t\right)$ propagates and is scattered by the surface of the target object, and some of the energy is returned to the radar receiver. Thus, the echo signal $s_{r}(t)$ could be expressed as Equation (2).

(2) $$s_{r}\left(t\right)=a_{1}exp\left[j2\pi \left(f_{c}\left(t+\tau \right)+\frac{1}{2}k{\left(t+\tau \right)}^{2}\right)\right]$$

where the time delay of propagation is τ=2R/c; R denotes the distance between the target and radar, and c denotes the light speed. After receiving the echo $s_{r}\left(t\right)$, the FMCW radar mixes it with $s_{t}\left(t\right)$ and applies low-pass filtering to obtain the beat signal $s_{b}(t)$, as shown in Equation (3).

(3) $$s_{b}\left(t\right)=a_{0}a_{1}exp\left[j2\pi \left(\frac{2kR^{2}}{c^{2}}-\frac{2f_{c}R}{c}-\frac{2ktR}{c}\right)\right]$$

It can be seen that among the three terms in Equation (3), only $\frac{2ktR}{c}$ contains a time-dependent frequency component.

The beat frequency ∆f bears a proportionality to the distance R, that is, $∆f\propto \frac{B}{cT_{m}}R$. The other two terms are phase components, which also scale linearly with the distance R.

**Figure 2:**
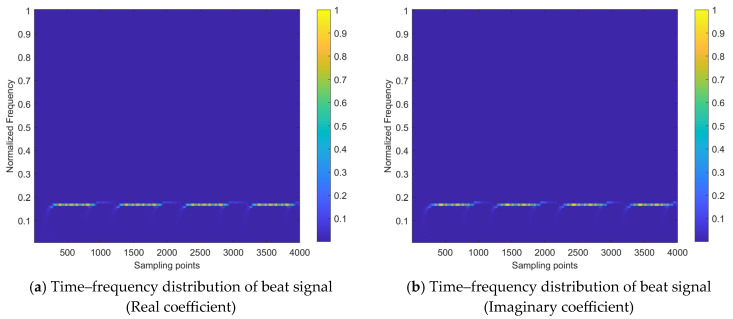

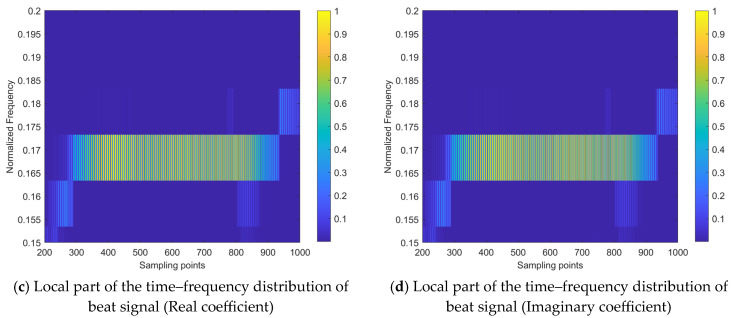
Beat signal time–frequency distribution image of target echo signal received by FMCW radar.

By applying the time–frequency transform to the beat signal $s_{b}\left(t\right)$, we can obtain a time–frequency distribution that is expressed as a matrix of coefficients. The normalized version of this distribution is shown in Figure 2. Specifically, Figure 2a,b display the real and imaginary parts of the coefficient matrix, respectively. Additionally, Figure 2c,d show the local part of the real and imaginary coefficients and the variation within a single modulation period. The *x*-axis represents the number of sampling points along the time variation, and the *y*-axis represents the normalized frequency spectrum.

In the battlefield environment, the FMCW radar may be affected by noise blanket interference and the periodic modulation interference radiated by electronic jammers. This results in the beat signal being contaminated by the interference components, making the target echo signal components insignificant, so the radar fails to correctly extract the target information. Without loss of generality, take two common examples for illustration, including a noise frequency-modulated (NFM) signal $J_{NFM}\left(t\right)$ and a sinusoidal frequency-modulated (SFM) signal $J_{SFM}\left(t\right)$. They can be defined uniformly as follows:

(4) $$J\left(t\right)=U_{0}+\exp\left[j2\pi \left(f_{j}t+\phi \left(t\right)\right)\right]$$

where $J\left(t\right)$ denotes the NFM signal $J_{NFM}\left(t\right)$ when $\phi \left(t\right)=k_{FM}\int_{0}^{t}u_{n}\left(\tau \right)d\tau$ and $J\left(t\right)$ denote the SFM signal, $J_{SFM}\left(t\right)$ when $\phi \left(t\right)=m_{f}sin\left(2\pi f_{m}t\right)$; $U_{0}$ denotes the amplitude of carrier signal; $f_{j}$ denotes the center frequency; $k_{FM}$ denotes the chirp rate; $u_{n}\left(t\right)$ denotes the noise, which is generally a stationary random process with a mean of 0 and a variance of $\sigma_{n}^{2}$; $m_{f}$ denotes the modulation index, and $f_{m}$ denotes the frequency of the sinusoidal modulated signal.

The interference signal enters the radar receiver and participates in the signal processing flow. The corresponding beat signal obtained by mixing and low-pass filtering can be expressed as:

(5) $$S_{b}^{J}\left(t\right)=a_{0}U_{0}exp\left[j2\pi \left(∆ft+\frac{1}{2}kt^{2}-\phi \left(t\right)\right)\right]$$

where ∆f denotes the center frequency difference between $J\left(t\right)$ and $s_{t}\left(t\right)$. When the center frequency of $J\left(t\right)$ approaches $s_{t}\left(t\right)$, the phase interference caused by $\phi \left(t\right)$ will be confused with the phase of $s_{b}\left(t\right)$, which leads to extraction errors in target information.

**Figure 3:**
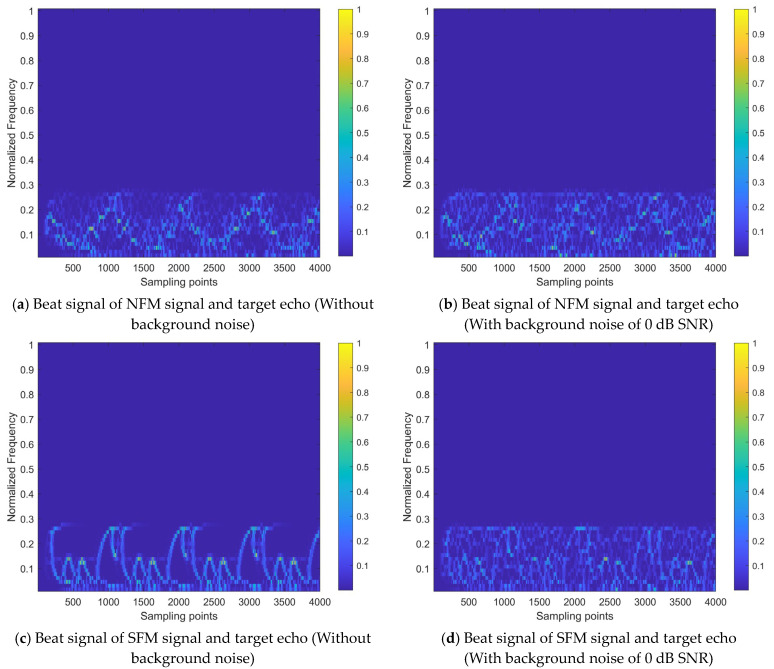
Beat signal time–frequency distribution containing both interference and target echo signal.

Take the interference-to-signal ratio (ISR) as 5 dB and the signal-to-noise ratio (SNR) of background noise as 0 dB. The time–frequency distribution image of $S_{b}^{J}\left(t\right)$ is shown in Figure 3.

By comparing Figure 2 with Figure 3, it can be observed that the interference signals and noise significantly reduce the recognizability of the target echo component within the received signal. The time–frequency energy distribution of the signal becomes disordered, leading to the inability of the FMCW radar to accurately extract target information and even to distinguish whether the target echo signal is present.

The interference mitigation task addressed in this paper can be regarded as a time–frequency distribution image restoration task. It involves restoring the time–frequency distribution state affected by interference, as shown in Figure 3, to the state with significant target echo components, as shown in Figure 2. This restoration process includes two parts: the removal of interference components and the recovery of target echo components.

Typical image restoration can be achieved by processing the image in its spatial domain or transform domain. In the spatial domain approach, common techniques such as mean filtering, median filtering, and Gaussian filtering correct the abnormal areas caused by interference by utilizing the spatial neighborhood information of pixels. Bilateral filtering uses the means of optimized dynamic weighting, which can avoid image blurring and thus preserve more details. However, the calculation of dynamic weights is more substantial and the parameter adjustment of filtering is complex. On the other hand, transform domain-based methods transform the image from the original spatial domain to a specific feature domain, where the abnormal areas exhibit distinct distribution characteristics. The selection strategy of the threshold is based on the distribution characteristics of anomalies, such as Visu-shrink, Bayes-shrink, and Sure-shrink.

Image restoration based on spatial domain or transform domain each have their advantages. The spatial domain-based method adjusts the pixel value of abnormal areas based on the information from neighboring areas, while the transform domain-based method is generally about the design of matching rules based on the characteristics of image anomalies. Due to the variability and non-stationarity of the interference signal, their morphological characteristics in the time–frequency distribution image undergo significant changes. This makes it difficult for matching rules to fit accurately in the transform domain. It also imposes certain limitations on the selection and utilization of neighborhoods for anomalies in the spatial domain. Ultimately, it leads to unsatisfactory image restoration and results in a single feature domain. Therefore, it is possible to combine approaches of both the spatial domain and transform domain for image restoration, fully leveraging the transform domain-based characteristics of interference areas and the information of neighboring areas around the interference areas in the spatial domain, to complement each other’s strengths, thus achieving more accurate image restoration. In the next section, a method using deep convolutional neural networks to automatically learn the characteristics of the transform domain for interference components and the neighboring areas’ information utilization of spatial domain will be proposed, thereby achieving better interference restoration effects.

## 3. Interference Mitigation Method

### 3.1. Interference Mitigation Procedure

For the received signals of FMCW radar, traditional interference mitigation strategies generally involve setting the signal strength to zero during the time periods or frequency bands where interference is present, which effectively avoids the impact of interference signals. However, when target echo signals coexist with interference, the zeroing operation inevitably affects the radar’s ability to discriminate targets and extract information, such as target range and velocity. The method proposed in this paper for interference mitigation aims to maximize the preservation of target echo signal components, while removing the interference signal components, ensuring that subsequent signal processing is unaffected. The process of this method is as follows:

(1) Perform time–frequency transform on the beat signal to obtain the time–frequency distribution, which includes the real-valued coefficient matrix and the imaginary-valued coefficient matrix. The time–frequency transform is implemented using the Fourier synchrosqueezed transform (FSST), with the rationale provided in Section 3.2;
(2) Apply the filtering algorithm proposed in Section 3.3 to the real-valued and imaginary-valued coefficient matrices of time–frequency distribution, respectively, to filter out the interference signal components contained in the two matrices and restore the strength of the target echo signal components;
(3) Apply the inverse FSST to the filtered results to convert the beat signal from the time–frequency domain back to the time domain and send it to the subsequent signal processing stages to complete the extraction of target information;

The core of the proposed interference mitigation method is the time–frequency distribution filtering algorithm. It treats the two time–frequency distribution coefficient matrices as image data for processing, which can be considered a type of adaptive time–frequency distribution filter.

### 3.2. Implement Approach of Time–Frequency Analysis

Time–frequency analysis is a signal analysis method that integrates characteristics from both the time and frequency domains. This approach decomposes signals into joint time–frequency functions, revealing the energy distribution of the signal across different temporal and spectral scales. It provides a comprehensive reflection of the signal’s properties, uncovering minute variations in the signal’s behavior across various time and frequency scales. Therefore, through time–frequency analysis, the characteristics of FMCW radar signals and interference signals can be more clearly and completely described, which is conducive to the identification and mitigation of interference signal components.

Short-time Fourier transform (STFT) is a typical method of time–frequency analysis. It cuts a signal sequence into several slices by a time window function g(t) and performs the Fourier transform to each slice to get the corresponding frequency. For a signal $x\left(t\right)$, its STFT is defined as Equation (6).

(6) $$X_{STFT}(t,f)=\int_{-\infty }^{\infty }x\left(t\right)g\left(t-mT\right)e^{-j2\pi ft}dt$$

where T denotes the length of w(t), m denotes the index of the slice. The STFT slides the window across the signal and computes the Fourier transform of the signal in each windowed segment. The result is a time–frequency representation of the signal, where the magnitude of $X_{STFT}(t,f)$ at each point (t,f) represents the amount of that frequency component present at that particular time. Common time window functions include the Hann window, Hamming window, Gaussian window, and Kaiser window, etc. There is a balance relationship between frequency resolution ∆f and time resolution ∆t, obtained by STFT due to the window function, which is called the uncertainty principle, shown as Equation (7). Therefore, it is impossible for both to take smaller values at the same time.

(7) $$∆f\cdot ∆t\geq \frac{1}{2\pi }$$

To avoid the limitations of the uncertainty principle, the means of post-processing could be taken for the time–frequency distribution obtained from STFT to achieve the energy reassignment, thereby improving the time–frequency resolution. Synchrosqueezed transform (SST) is a typical post-processing-based time–frequency transform method [44]. It includes two implementations, namely frequency-domain-based reassignment and time-domain-based reassignment. Fourier synchrosqueezed transform (FSST) is defined as the frequency-domain-based reassignment transform based on STFT. It is defined as Equation (8).

(8) $$\begin{array}{c} S_{x}^{g}(t,\omega )=\int_{-\infty }^{\infty }\;x(\tau )g^{*}(\tau -t)e^{-j\omega (\tau -t)}d\tau =\frac{1}{2\pi }\int_{-\infty }^{\infty }\;X(v)G^{*}(v-\omega )e^{ivt}dv \end{array}$$

where ω denotes the instantaneous angular frequency, τ denotes the time variable; $X\left(v\right)$ is the frequency domain representation of the signal $x\left(t\right)$ and $G\left(\omega \right)$ is the frequency domain representation of the window function g(t). FSST performs the frequency-domain-based reassignment by the estimated value $\hat{\omega }(t,\omega )$ of the instantaneous frequency operator (IFO). The first-order expression for IFO is shown as Equation (9).

(9) $$\hat{\omega }(t,\omega )=R\left(\frac{1}{2\pi j}\frac{\partial_{t}S_{x}^{g}(t,\omega )}{S_{x}^{g}(t,\omega )}\right)$$

where R(·) denotes taking the real value of a complex number. Thus, FSST and its inverse transform can be expressed as:

(10) $$T_{f}(t,\omega )=\frac{1}{2\pi g^{*}(0)}\int_{-\infty }^{\infty }S_{x}^{g}(t,\omega )\delta (\omega -\hat{\omega }(t,v))dv$$

(11) $$x(t)=\frac{1}{2\pi g^{*}(0)}\int_{-\infty }^{\infty }S_{x}^{g}(t,\omega )d\omega$$

FSST inherently provides strong robustness against noise due to its signal energy processing approach. Additionally, it meets other requirements for the FMCW radar interference mitigation task in time–frequency transform methods, including the ability to maximally differentiate signal components with frequency distribution differences and to satisfy the requirements for computational efficiency and transform reversibility. Therefore, we apply FSST to interference mitigation.

### 3.3. Filtering Algorithm for Time–Frequency Distribution

Assuming that the time–frequency distribution of the received signal of FMCW radar containing only the target echo is denoted as x, and the one containing both the target echo and interference is denoted as y, then interference mitigation from the Bayesian probability perspective can be described as:

(12) $$P_{X|Y}(x|y)=\frac{P_{XY}(x,y)}{P_{Y}(y)}=\frac{P_{Y|X}(y|x)P_{X}(x)}{P_{Y}(y)}$$

where X and Y are the conditional variables of x and y, respectively; $P_{X}(x)$ and $P_{Y}(y)$ are the probability distribution of x and y, respectively. The goal of interference mitigation is to find the maximum a posteriori (MAP) estimate $\hat{x}$ of the pure target echo signal x, that is:

(13) $$\hat{x}=\arg\underset{x}{max}P_{X|Y}(x|y)$$

For the image restoration task, the typical supervised deep learning approach involves learning the inverse process of the interference effect from a given input $P_{Y}(y)$ and a ground truth output $P_{X}(x)$. The goal is to estimate the inverse probability $P_{X|Y}(x|y)$, which allows for interference mitigation in any given y and the minimization of the estimate error to obtain $\hat{x}$.

Inspired by the principles of image damage and image restoration, this paper proposes an a priori assumption that any interference can be decomposed into a multiplicative interference component $n_{a}\left(x\right)$ and an additive interference component $n_{b}\left(x\right)$. The multiplicative interference component can be attributed to the non-stationary dynamic characteristics of the signal, or the effects of complex propagation environments on the channel, while the additive interference component corresponds to factors that are independent in terms of probability distribution. Therefore, the time–frequency distribution image y of the target echo signal with interference can be expressed as:

(14) $$y=x\cdot n_{a}(x)+n_{b}(x)$$

If the estimated values of the above two types of interference components are set to ${\hat{n}}_{a}(x)$ and ${\hat{n}}_{b}(x)$, then interference mitigation can be achieved by:

(15) $$\hat{x}=\frac{y-{\hat{n}}_{b}(x)}{{\hat{n}}_{a}(x)}$$

**Figure 4:**
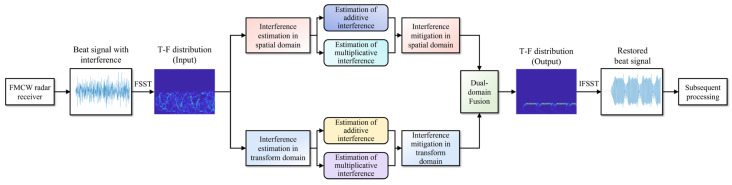
Schematic diagram of the proposed interference mitigation method.

Based on the aforementioned a priori assumption and interference mitigation approach, this paper proposes a dual-domain fusion filtering algorithm for the time–frequency distribution image of the beat signal, to achieve adaptive mitigation of various types of non-coherent interference. The algorithm employs specially designed convolutional neural network (CNN) models in both the spatial domain and the transform domain of the time–frequency distribution image to obtain ${\hat{n}}_{a}(x)$ and ${\hat{n}}_{b}(x)$, respectively. It then realizes interference mitigation in the single domain through Equation (15). Finally, the interference mitigation results from the two domains are fused to obtain the final outcome, which can be further transformed into the time domain expression for subsequent processing. Figure 4 illustrates the whole process flow of the proposed interference mitigation method. Each step involved will be detailed in the subsequent sections.

#### 3.3.1. Spatial Domain-Based Interference Estimate and Mitigation

(1) Overall Structure

The spatial domain interference estimation module is implemented based on the improved neural network structure of UNet++. UNet++ is a convolutional network structure designed for pixel-level tasks. It is an optimized version of the image segmentation model UNet, featuring dense skip connections between the encoder and decoder modules, and it incorporates the sub-networks within the main trunk of the encoder–decoder structure. This enhances its ability to extract the target’s positional features and semantic features [45].

**Figure 5:**
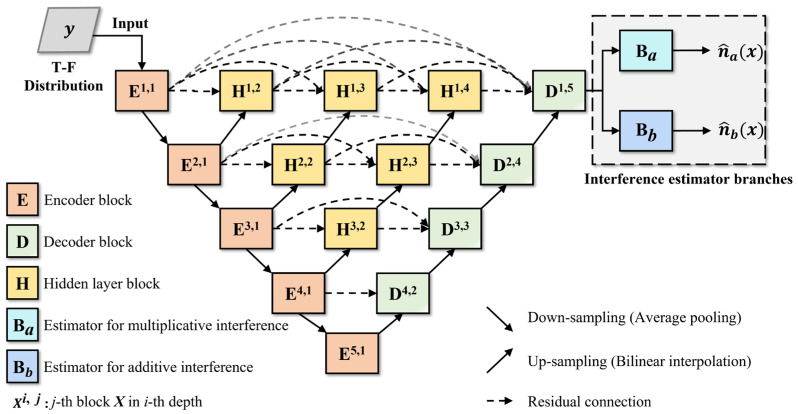
Structure diagram of spatial domain interference estimation module.

Figure 5 illustrates the structure of the spatial domain interference estimation module, where the improved UNet++ structure serves as the spatial domain feature extraction module. It detects and extracts the interference components from the time–frequency distribution image, then uses the interference estimator branches to obtain the estimates for both additive and multiplicative interference components.

As shown in Figure 5, the feature extraction module of UNet++ includes encoder blocks, decoder blocks, and hidden layer blocks. Through the collaboration of the multi-level encoder and decoder blocks, the localization features and semantic features of interference components at different scales within the time–frequency image can be extracted. The hidden layer blocks are located between the encoder and decoder blocks, and a hidden layer block, at a certain level, receives the output from the same level module and a deeper level block. By combining this with residual connections within the same layer, it achieves feature aggregation across different scales, thereby enhancing the identification capability for interference features.

(2) RGC Module

**Figure 6:**
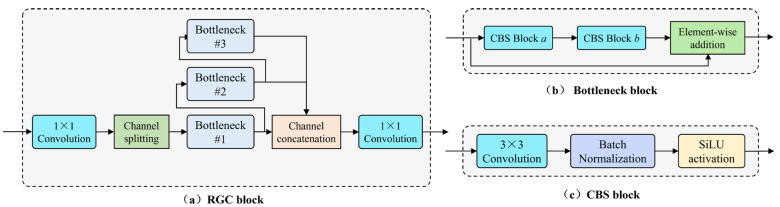
Structure diagram of RGC module.

To further enhance the ability to extract pixel-wise feature differences, we introduce the rich gradient-flow convolution (RGC) module to replace the original double-layer convolutional module used in UNet++. It serves as the implementation of the encoder block, decoder block, and hidden layer block. Its structure is shown in Figure 6.

The RGC module is designed to improve the ability to extract features with a small number of parameters by reducing redundant gradient information and aggregating features under different sizes of receptive fields. For UNet-like networks that contain hierarchical down-sampling and up-sampling operations, the deeper the layer of the encoder or decoder module, the smaller the scale of the feature map and the greater the number of channels. Although this ensures the extraction of enough feature representations of abnormal areas, it also inevitably leads to redundant gradients during model training. To reduce redundant computational load, the RGC module performs channel splitting operations to take only half of the number of channels from the feature map at one time, then uses stacked bottleneck module groups for feature extraction. The bottleneck module consists of two CBS modules (including convolution, batch normalization, and SiLU activation), where the first CBS module halves the number of input feature map channels, and the second CBS module restores it, with a residual connection added afterward. The CBS module employs the Sigmoid-weighted linear unit (SiLU) activation function to provide the capability for non-linear mapping, which is defined as follows:

(16) $$F_{\mathrm{SiLU}}(x)=x\cdot F_{\mathrm{Sigmoid}}(x)=\frac{x}{1+e^{-x}}$$

Additionally, in the stacked bottleneck structure of the RGC module, the number of bottleneck modules that the feature maps pass through increases progressively, serving the purpose of extracting target feature representations under different receptive field scopes within the same scale feature maps. Then, the outputs from multiple bottleneck modules are fused across levels by element-wise addition. Finally, the fusion result is channel-wisely concatenated with the other half of the feature maps that did not enter the stacked bottleneck modules and then outputted. In this process, the stacking operation plays the role of sharing convolutional kernel parameters. During the backpropagation stage for updating the convolutional kernel parameters’ weights, bottleneck module #1 has an increased number of gradient flow paths compared to when there is no stacked structure, due to the additional sources from the second and third bottleneck modules. Hence, it is referred to as the rich gradient-flow convolution module. It should be noted that the 1 × 1 convolutional layers placed at the head and tail of the module are used to match the number of feature map channels set at different depth levels of the model.

(3) Dimensional Attention Mechanism

The morphological characteristics of different interference types in the time–frequency image vary; some are point-like, bar-like (horizontal or vertical), or block-like, etc. Targeted feature enhancement, based on their morphological characteristics, will aid in the extraction of differentiated features. Therefore, two branches, $B_{a}$ and $B_{b}$, are added to the tail of the UNet++ structure. These branches are used to obtain estimates, ${\hat{n}}_{a}(x)$ and ${\hat{n}}_{b}\left(x\right)$, for the additive and multiplicative interference components, respectively, from the output of feature maps by the UNet++ structure. Both estimator branches, $B_{a}$ and $B_{b}$, consist of three cascaded dimensional attention modules of different dimensions (i.e., height dimension H, width dimension W, and channel dimension C). During model training, the two branches differentiate their functional specificity by updating the parameter weight. The dimensional attention module applies attention feature enhancement to a certain dimension of the feature map, based on the proposed dimensional attention mechanism. The purpose of using the attention mechanism is to simulate the working principles of the human visual system, to weight different regions of the feature map that contain various content and enable the neural network to focus more on important features, thereby improving the accuracy and efficiency of image feature recognition and processing.

**Figure 7:**
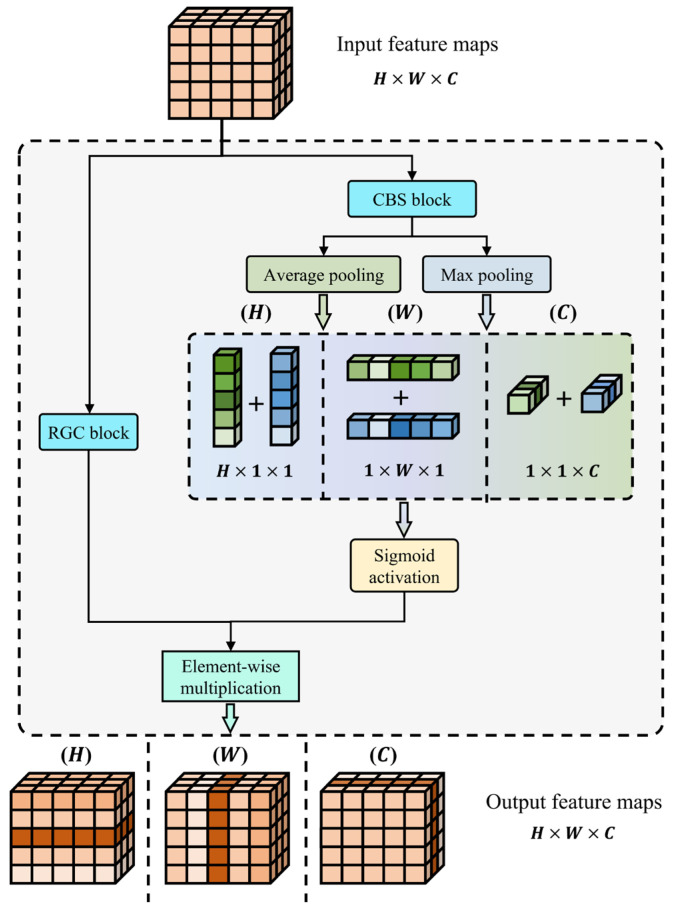
Schematic diagram of dimensional attention mechanism.

The proposed dimensional attention mechanism, as shown in Figure 7, is structurally divided into a main trunk and an attention score branch. The feature maps and attention scores obtained from these two branches are multiplied, by way of element-wise, to produce the attention-weighted feature map as the output. Equation (17) illustrates the generation approach of the attention score when taking the H dimension as an example. A feature map x of shape H⁡×W⁡×C is processed through a CBS module to obtain $x_{b}$, then subjected to both average pooling and max pooling to generate feature maps $f_{avg}^{h}(x_{b})$ and $f_{max}^{h}(x_{b})$ of shape H⁡×1×1, respectively. Finally, the sum of these two is activated by a Sigmoid function to obtain the attention score $\omega^{h}(x)$ of height dimension H.

(17) $$\omega^{h}(x)=F_{Sigmoid}\left[f_{avg}^{h}(x_{b})+f_{max}^{h}(x_{b})\right]$$

After obtaining ${\hat{n}}_{a}(x)$ and ${\hat{n}}_{b}(x)$ from the interference estimator branches, it is possible to implement spatial domain-based interference mitigation for the time–frequency distribution image y of the interfered echo signal through Equation (15), thereby obtaining the corresponding result ${\hat{x}}_{s}$.

#### 3.3.2. Transform Domain-Based Interference Estimate and Mitigation

**Figure 8:**
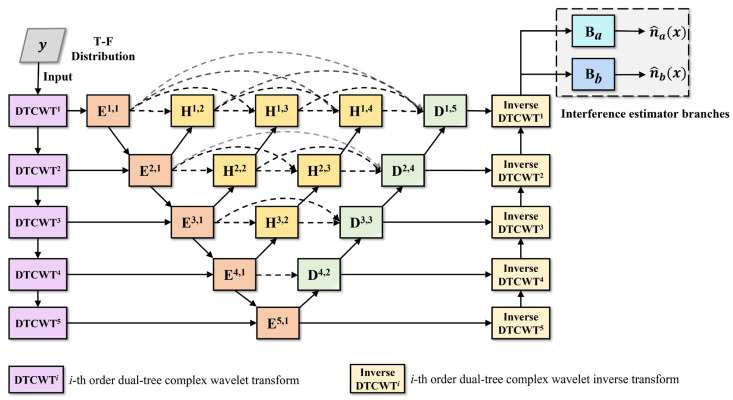
Structure diagram of transform domain interference estimation module.

The transform domain interference estimation module is implemented by integrating dual-tree complex wavelet transform (DTCWT) with the improved UNet++ structure and the interference estimator branch, as shown in Figure 8. The module first converts the image from the spatial domain to the transform domain, using the five-order DTCWT. It then recognizes and extracts the interference components through the improved UNet++ structure. Finally, it obtains the corresponding spatial domain representation through the inverse DTCWT and the interference estimates through the estimator branches.

The DTCWT performs multi-stage discrete wavelet transforms on the time–frequency image, along the branches of the real-valued part and imaginary-valued part, achieving the diverse characteristic component decomposition at different scales. The resulting complex wavelet coefficients are as follows:

(18) $$f_{m,n}^{\theta }(x)=p_{m,n}^{\theta }(x)+i\cdot q_{m,n}^{\theta }(x)$$

where $f_{m,n}^{\theta }$ denotes the wavelet coefficient at position (m,n) on the complex plane at the θ-th layer, m denotes the spatial position within the sub-band and n denotes the temporal position of the tree node; $p_{m,n}^{\theta }$ and $q_{m,n}^{\theta }$ are the real-valued part coefficient and the imaginary-valued part coefficient, respectively, defined as follows:

(19) $$p_{m,n}^{\theta }(x)=\sum_{k=0}^{L-1}h_{k}^{\theta }(x-2n)\cdot p_{2m-k,n}^{\theta -1}(x)$$

(20) $$q_{m,n}^{\theta }(x)=\sum_{k=0}^{L-1}g_{k}^{\theta }(x-2n)\cdot p_{2m-k,n}^{\theta -1}(x)$$

where $h_{k}^{\theta }$ and $g_{k}^{\theta }$ denote the coefficients of low-pass and high-pass filter, respectively; $p_{2m-k,n}^{\theta -1}(x)$ denotes the real-valued part wavelet coefficient for the node of 2m−k in the layer of θ−1.

**Figure 9:**
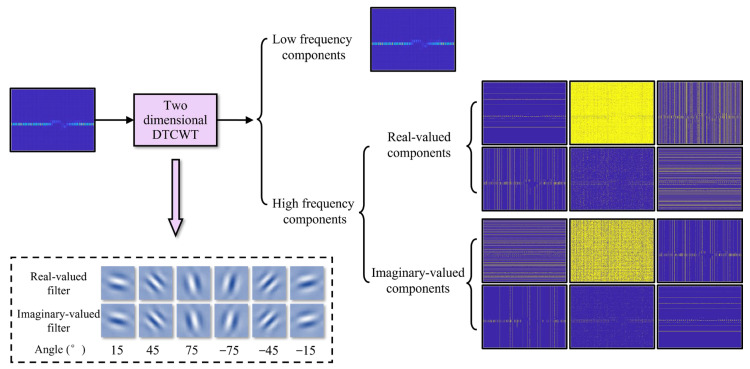
Principle diagram of two-dimensional dual-tree complex wavelet transform of beat signal time–frequency distribution image.

The real-valued branch of the DTCWT serves to extract the texture information of the image, while the imaginary-valued branch serves to extract the structural information, such as edges and contours. As shown in Figure 9, each level of decomposition consists of the low-frequency detail, as well as six high-frequency details in six directions of ±15 degrees, ±45 degrees, and ±75 degrees. Compared to the image wavelet transform (IWT), which only provides information in three directions, horizontal, vertical, and diagonal, DTCWT has better characteristics of directional selectivity, translation invariance, and multi-resolution, allowing it to extract more information from the image [46]. Therefore, DTCWT helps to enhance the ability to separate and extract high-frequency anomaly areas from the low-frequency image background across different scales and directions.

In the transform domain interference estimation module shown in Figure 8, the time–frequency distribution image is first subjected to a multi-stage DTCWT to achieve the transition from the spatial domain to the transform domain and to preliminarily separate the interference components at different scales. Then, further feature extraction is performed through the encoder and decoder modules at each level. For the encoder and decoder modules, across stages, down-sampling and up-sampling methods are used to promote the fusion of interference feature information across scales, thus enhancing the feature extraction capability. Finally, the spatial domain representation of the interference component features is obtained through inverse DTCWT.

It should be noted that the encoder and decoder modules are also implemented by the RGC module. Since interference and noise generally correspond to the high-frequency components of the time–frequency image, the encoder and decoder modules only process the high-frequency components output by the DTCWT, to reduce redundant computational load.

In addition, since the interference mitigation approach, based on the a priori assumption shown in Equation (15), is the expression based on the spatial domain, the extracted interference components contained in the feature map need to be transformed back to the spatial domain through inverse DTCWT. The result of the higher-order inverse DTCWT is used as the input for the lower-order inverse DTCWT, and the result of the first order inverse DTCWT is used as the input for the interference estimator branches. The structure of the interference estimator branch in the transform domain is consistent with that of the spatial domain. Finally, the interference mitigation for this domain is also performed through Equation (15), to obtain the corresponding result ${\hat{x}}_{t}$.

#### 3.3.3. Dual-Domain Fusion Interference Mitigation

Due to the variability of interference types and the fluctuations of interference signal strength, their expression in the time–frequency distribution image also changes dynamically. Consequently, the contributions of the interference mitigation results, based on the spatial domain and transform domain to the final interference mitigation fusion result, will also vary accordingly. A simple arithmetic mean approach cannot perceive the relative quality of the interference mitigation results in the two domains, nor does it enable the corresponding weight allocation. This will result in the loss of the better interference mitigation effect of one certain domain, thus reducing the overall interference mitigation effect.

**Figure 10:**
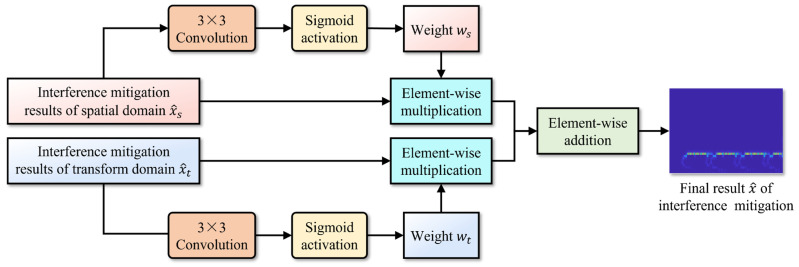
Schematic diagram of dual-domain fusion module.

The dual-domain fusion module, as illustrated in Figure 10, is proposed as a solution to the aforementioned problem. It employs an adaptive weight generation process, based on the single domain interference mitigation result, thus promoting better fusion results. Specifically, the single domain interference mitigation result is processed through a 3 × 3 convolutional layer and the sigmoid activation, to obtain a pixel-wise weight that is consistent with the interference mitigation result in shape and has values ranging between 0 and 1. Finally, the generated weights are used to perform element-wise multiplication with the dual-domain interference mitigation results, followed by the element-wise addition between the weighted interference mitigation results, thereby obtaining the final result of interference mitigation for the time–frequency image.

## 4. Simulation Experiment

### 4.1. Configurations of Model Structure and Model Training

In general, the scale of a neural network model has a marginal effect on its performance for a particular task. Thus, we finally determined the following structure configurations of the proposed neural network model, after the comprehensive consideration of the time–frequency image resolution and the performance test results of the model structure

(1) For the UNet++ structure, serving as the interference estimation module in both domains, its layer depth is set to 5, with the number of feature map channels used for feature extraction in the first to fifth level layer being 4, 16, 32, 64, and 64, respectively;
(2) The outputs of the interference estimator branches in both domains, namely the additive and multiplicative interference component estimates, are all 1-channel feature maps.

The loss function for model training employs the Smooth L1 function, also known as the Huber loss, which is defined as Equation (21).

(21) $$L_{\mathrm{Smooth}\;L1}(x_{i},y_{i})=\left\{\begin{array}{c} 0.5(x_{i}-y_{i}{)}^{2},\;\;\;\;|x_{i}-y_{i}|<1\\ \left|x_{i}-y_{i}\right|-0.5,\;\;\;\;|x_{i}-y_{i}|\geq 1 \end{array}\right.$$

where i denotes the index of the current pixel; x and y denote the restored results of the time–frequency image output by the proposed algorithm and the ideal restoration effect, respectively. Both are also referred to as the prediction and ground truth (GT). This loss function incorporates the advantages of both the mean absolute error (MAE) and the mean square error (MSE), meaning that the function is smooth and differentiable everywhere, while having a stable gradient, which prevents gradient explosion.

Additionally, the upper limit of the training epoch is set to 300; the training batch size is 2; the optimization algorithm is AdamW, with a weight decay factor being $1\times 10^{-4}$; the initial learning rate is 0.001, and the learning rate update strategy is step decay with a decay factor of 0.65 and a decay period of 80 training epochs. The training platform is RTX 3080 and the software environment is PyTorch 2.0.1.

### 4.2. Datasets and Preparation Details

The signal datasets required for neural network training and evaluation are synthesized by simulation. Table 1 provides the parameter configuration for signal simulation and time–frequency analysis. To ensure reliable reproduction of interference across various scenarios, some parameters are obtained through random sampling based on a uniform distribution within a given range. In addition, the simulation also takes into account the impact of background Gaussian noise.

The process for the dataset synthesis is as follows:

(1) Synthesize LFM signals through simulation to serve as the FMCW radar transmission signals, with certain phase delay forms set as the target echo signals;
(2) Synthesize interference signals by simulation, including NFM signal, SFM signal, and swept-frequency signal;
(3) Superimpose each type of interference signal onto the target echo signal;
(4) Add broadband Gaussian white noise to simulate background noise, then obtain the received signals for FMCW radar;
(5) The received signals are mixed with a local reference signal and then low-pass filtered to obtain the beat signal;
(6) Perform the time–frequency transform of FSST for the beat signal and obtain the corresponding time–frequency distribution image samples. Among these, samples that contain only the target echo serve as the ground truth for model training and evaluation, while samples with various interferences and noise are used as the input for the proposed algorithm.

In the dataset, there are 1000 samples for each type of interference, with 800 used as the training set and the remaining 200 as the validation set. The time–frequency transform results include two parts; the real-valued coefficient and the imaginary-valued coefficient, corresponding to two time–frequency distribution images for each signal sample. Therefore, a complete interference mitigation process involves the interference mitigation for both the real-valued and imaginary-valued coefficients.

### 4.3. Computational Complexity Evaluation

It is necessary to analyze the computational complexity of the neural network model to evaluate the model’s operational efficiency and the demand for computational resources. The computational complexity of neural networks is typically assessed using two metrics, params and FLOPs. Params refer to the number of weight parameters that can be updated through model training, FLOPs refer to the total number of floating-point operations performed in the model. For convolutional neural network models, the calculation methods for params and FLOPs in the convolutional layers are as follows:

(22) $$F_{\mathrm{conv}}^{\mathrm{Params}}=\left(k_{w}\times k_{h}\times c_{in}\right)\times c_{out}+c_{out}$$

(23) $$F_{\mathrm{conv}}^{\mathrm{FLOPs}}=\left({2\times k}_{w}\times k_{h}\times c_{in}\times c_{out}+c_{out}\right)\times f_{w}\times f_{h}$$

where $k_{w}$ and $k_{h}$ denote the width and height of the convolutional kernel, respectively; $c_{in}$ and $c_{out}$ denote the number of input channels and output channels of the convolutional kernel, respectively; $f_{w}$ and $f_{h}$ denote the width and height of the feature map, respectively.

### 4.4. Performance Evaluation Metrics

#### 4.4.1. Evaluation of Image Restoration

For the image restoration task, the peak signal-to-noise ratio (PSNR) and structural similarity (SSIM) are commonly used to evaluate the restoration and recovery effects on the image.

PSNR is a commonly used metric in the fields of image reconstruction. It represents the ratio between the maximum possible power $P_{max}$ of the image and the power $Q_{n}$ of the noise that affects its fidelity, generally expressed on a decibel (dB) scale, that is:

(24) $$f_{PSNR}=10\cdot lg\left(\frac{P_{max}^{2}}{Q_{n}}\right)$$

where $P_{max}$ is 255 for the 8-bit valued image; $Q_{n}$ is defined as follows:

(25) $$Q_{n}\left(x,t\right)=\frac{1}{N}\sum_{p\in P}\;{\left[x\left(p\right)-t\left(p\right)\right]}^{2}$$

where x represents the image output from the neural network model; t represents the ground truth, p represents an arbitrary pixel point, P represents the set of all pixel points, and N denotes the size of P.

SSIM provides a measure of the similarity in terms of image luminance l, contrast c, and structural information s. Here, image luminance is represented by the mean of pixel values, contrast is represented by the standard deviation of pixel values, and structural information is represented by the correlation coefficient. For the prediction x and ground truth t, the definition of SSIM is as follows:

(26) $$f_{\mathrm{SSIM}}(x,t)=l(x,t)+c(x,t)+s(x,t)=\frac{\left(2\mu_{x}\mu_{t}+C_{1}\right)\left(2\sigma_{xt}+C_{2}\right)}{\left(\mu_{x}^{2}+\mu_{t}^{2}+C_{1}\right)\left(\sigma_{x}^{2}+\sigma_{t}^{2}+C_{2}\right)}$$

where $\mu_{x}$ and $\mu_{t}$ denote the mean of pixel values of x and t, respectively; $\sigma_{x}$ and $\sigma_{t}$ denote the standard deviation, $\sigma_{xt}$ denotes the covariance; $C_{1}$ and $C_{2}$ are constant coefficient, being (0.01 × 255)^2^ and (0.03 × 255)^2^, respectively.

The higher the values of PSNR and SSIM, the smaller the power of interference contained in the prediction, and the stronger the algorithm’s interference mitigation capability.

#### 4.4.2. Evaluation of Time–Frequency Energy Aggregation Degree

Rényi entropy (RE) is served for the energy aggregation degree evaluation of the restored time–frequency distribution image. It is a measure of uncertainty or randomness and can also be used to describe the complexity of probability distributions [47]. It is a generalization of Shannon entropy in information theory, with the calculation method adjusted by the parameter α, thus providing flexibility in addressing various scales of information measurement and statistical analyses. The smaller the value of RE, the higher the energy aggregation degree of the time–frequency distribution.

For a discrete random variable X, with its probability distribution given by $P\left(X\right)=\{p\left(x_{1}\right),p\left(x_{2}\right),\ldots ,p\left(x_{n}\right)\}$, the corresponding RE is defined as:

(27) $$R_{\alpha }\left(X\right)=\frac{1}{1-\alpha }\ln\left[\sum_{i=1}^{n}p{\left(x_{i}\right)}^{\alpha }\right]$$

where α>0 and α≠1; when α→1, $R_{\alpha }(X)$ degrades to the Shannon Entropy $H_{1}(X)$, as shown in Equation (28).

(28) $$H_{1}(X)\begin{array}{c} =\underset{\alpha \to 1}{\lim}\;\frac{1}{1-\alpha }\ln\;\left[\sum_{i=1}^{n}\;p{\left(x_{i}\right)}^{\alpha }\right] \end{array}=-\sum_{i=1}^{n}p\left(x_{i}\right)lnp\left(x_{i}\right)$$

For a time–frequency distribution image T(t,f), its RE could be defined as:

(29) $$R_{\alpha }(T)=\frac{1}{1-\alpha }\ln\frac{\iint T(t,f{)}^{a}dtdf}{\iint T(t,f)dtdf}$$

where t denotes the time variable, f denotes the frequency variable, α is set to 3.

#### 4.4.3. Evaluation of Signal Quality

Taking into account the impact of interference and noise on radar signal processing, the signal-to-interference-plus-noise ratio (SINR) serves as a metric to measure the strength of the relationship of the target echo signal to interference and noise within the radar received signal. SINR is generally expressed on a decibel (dB) scale and can be calculated as follows:

(30) $$\mathrm{SINR}=10\cdot \mathrm{lg}\frac{{\left|\left|s_{t}\right|\right|}_{2}^{2}}{{\left|\left|s_{x}-s_{t}\right|\right|}_{2}^{2}}$$

where $s_{t}$ denotes the radar received signal without interference and noise; $s_{x}$ denotes the radar received signal with interference and noise; ${\left|\left|\cdot \right|\right|}_{2}$ denotes the L2 norm operator of the signal sequence. Therefore, the improvement in signal quality brought by the interference mitigation can be evaluated quantitatively by comparing the SINR variation ∆SINR of the beat signals before and after the mitigation process, as shown in Equation (31).

(31) $$∆\mathrm{SINR}={\mathrm{SINR}}_{\mathrm{after}}-{\mathrm{SINR}}_{\mathrm{before}}$$

A specific interference signal corresponds to a specific ${\mathrm{SINR}\;}_{\mathrm{before}}$, thus, the greater the value of ∆SINR, the less that the strength of the interference and noise components remain in the beat signal after interference suppression, indicating that the interference suppression operation has a stronger ability to suppress interference and noise.

## 5. Results

### 5.1. Ablation Experiments

To verify the effectiveness of the proposed interference mitigation method based, on the a priori assumptions and the dual-domain fusion strategy, we provide the introduction and the analysis of the ablation experiments, with the interference mitigation task of the NFM signal as an example.

**Figure 11:**
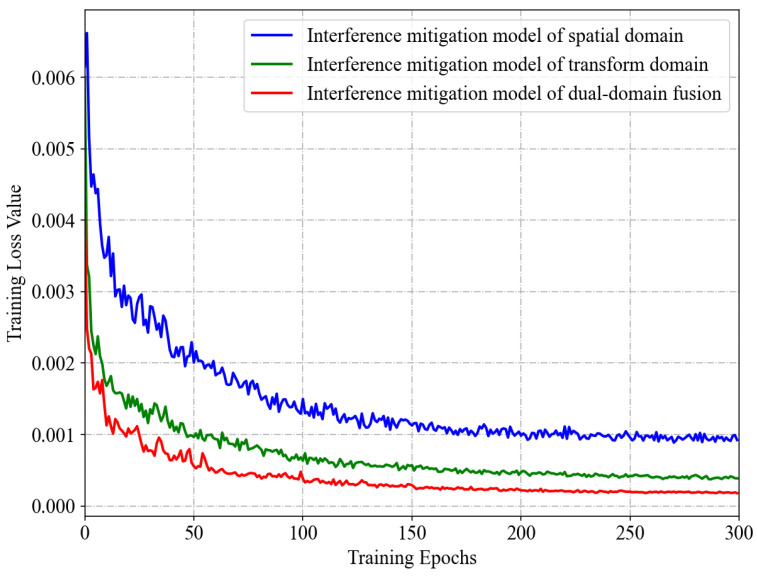
Training convergence effects of dual-domain fusion neural network model and single-domain neural network model.

Figure 11 presents a comparison of the convergence effects of the dual-domain fusion network model of interference mitigation versus the two single-domain network models, including the spatial domain model and the transform domain model. To ensure a fair comparison, the datasets and hyperparameter configurations used for training all three models are consistent.

It can be observed from Figure 11 that the training convergence rate (i.e., the descent rate of the loss function curve) of the dual-domain fusion network model is the fastest of the three, while the spatial domain model is the slowest. Furthermore, when the loss function values of the models have essentially stabilized, the dual-domain fusion model has the smallest loss function value, followed by the transform domain model and the spatial domain model. When a loss function benchmark is employed, the loss function value can be used to represent the relative performance trend of the models. Consequently, the above phenomena can be considered to demonstrate two characteristics of the proposed method. Firstly, the interference mitigation of the transform domain based on DTCWT has a more pronounced effect than the conventional approach of the spatial domain. Secondly, the fusion of interference mitigations from the two feature domains further enhances the interference mitigation effect.

The quantitative comparison and verification of the average interference mitigation performance of the three models on the validation set samples is provided in Table 2, which is based on the performance evaluation metrics introduced in Section 4.4. It should be noted that, for the metric RE of the time–frequency energy aggregation degree evaluation, a smaller metric value indicates better interference mitigation effects. For the metrics of image restoration and signal quality evaluation, a larger metric value indicates better interference mitigation effects. It can be observed that the order of the interference mitigation capabilities of the three models, from strongest to weakest, is as follows: the dual-domain fusion model, the transform domain model, and the spatial domain model.

**Figure 12:**
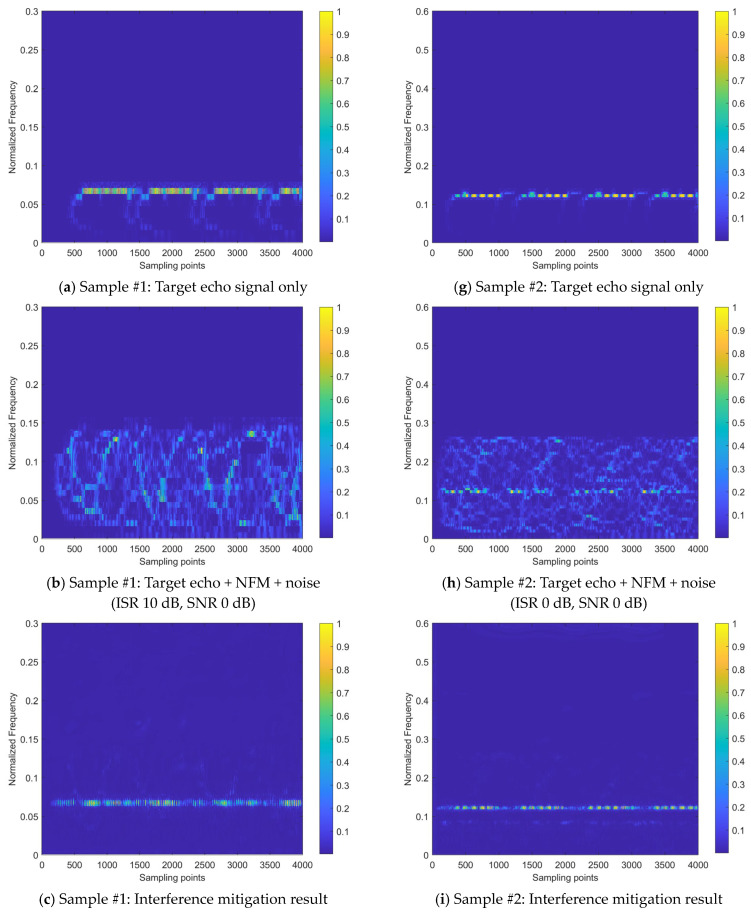

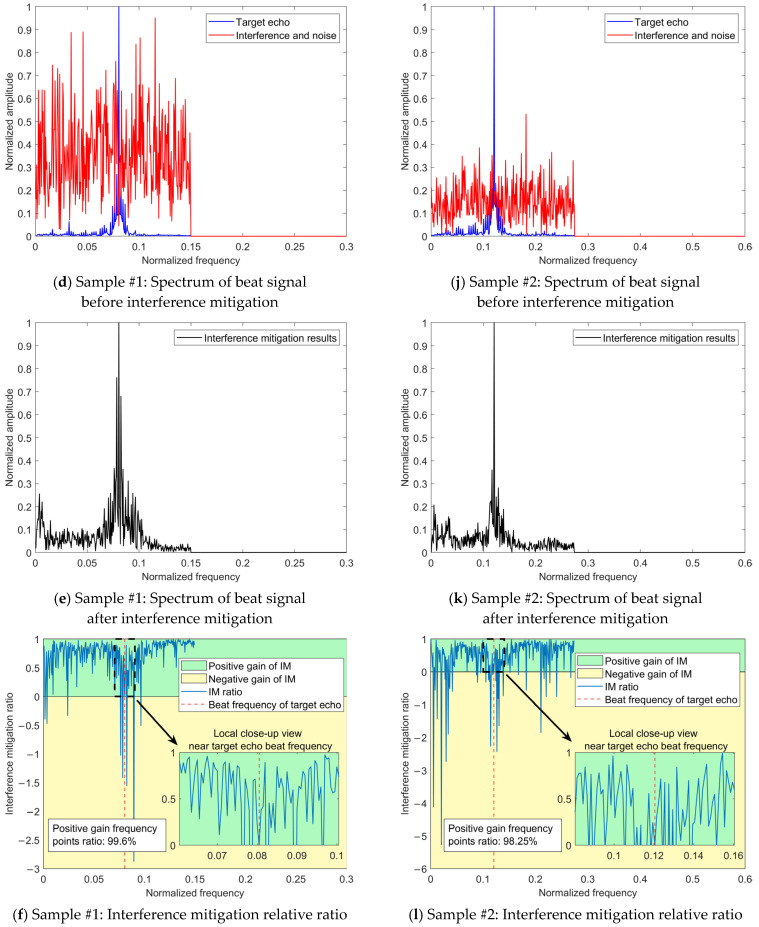
Interference mitigation effects on NFM signals of the proposed method.

Figure 12 presents the interference mitigation effects of the proposed method on the NFM interference signal by two samples. The figure displays six sub-figures per row for one signal sample. The first row sub-figure shows the time–frequency distribution of the beat signal for the target echo signal without noise and interference (taking the real-valued coefficient of the time–frequency distribution as an example); the second sub-figure row shows the time–frequency distribution of the beat signal after applying Gaussian noise with the SNR of 0 dB and two intensities of NFM interference signals, where signal sample #1 is subjected to an NFM interference signal with the ISR of 10 dB, and signal sample #2 is subjected to an NFM interference signal with the ISR of 0 dB; the third row sub-figure shows the results obtained after applying the proposed interference mitigation method to the interfered signal samples; the fourth sub-figure row shows the spectrums of radar beat signal before interference mitigation, including target echo, interference, and noise; the fifth row sub-figure shows the spectrum of radar beat signal after interference mitigation; the sixth row sub-figure shows the interference mitigation (IM) relative ratio, which is defined as the ratio between the strength variation of the beat signal spectrum brought by the interference mitigation and the strength of the beat signal spectrum before the interference mitigation. The green regions in the sub-figure denote the positive value of IM relative ratio, while the yellow regions denote the negative value of IM relative ratio. The positive gain frequency points ratio, labeled in the lower left corner of the sub-figure, refers to the ratio between the number of the frequency points with the positive IM relative ratios in the green regions to the number of total frequency points across the spectrum. This ratio, along with the collaboration of signal quality evaluation metric ∆SINR, provides the overall IM performance evaluation reference of the proposed method on a beat signal.

It can be observed from Figure 12 that the beat signal of the received signal is suppressed by interference and noise, leading to a decrease in the significance of the target echo signal component. This is particularly evident at the ISR of 10 dB, where the intensity of the target echo signal component is greatly suppressed by the interference signal. The time–frequency energy distribution is extensively and irregularly diffused to the noise and interference signal components, making it difficult to discern the presence of the target echo signal. After the application of the proposed interference mitigation method, the time–frequency distributions of both signal samples have been significantly improved. The majority of the interference and noise components within the low-pass filtering band have been removed. Moreover, the intensity of the target echo components is restored to some extent and the corresponding energy aggregation degree is improved as well. On the other hand, compared to the state of the time–frequency energy distribution without interference, although the majority of the interference and noise energy has been removed by the proposed method, there is still a certain degree of error in the restoration of the temporal distribution pattern of the target echo. This is particularly evident during the initial time stage of Sample #1.

In addition, Figure 12 also shows the spectrums of the radar beat signal before and after interference mitigation. The beat signal after interference mitigation was obtained through the inverse FSST using the real and imaginary coefficients of the time–frequency distributions. It can be observed that the intensity of noise and interference signal has been significantly reduced through the proposed interference suppression method. Regarding the IM relative ratio based on the beat signal spectrum, the ratio values of the major frequency points are between 0 and 1, indicating the expected IM effects. A small number of frequency points correspond to a negative ratio value, located in the yellow-marked area of negative gain of IM in the figure, which suggests that the proposed method has not achieved recognition of interference and noise at this frequency point and has amplified their intensity. This indicates that there is still room for improvement in the method’s ability to identify interference and noise.

**Figure 13:**
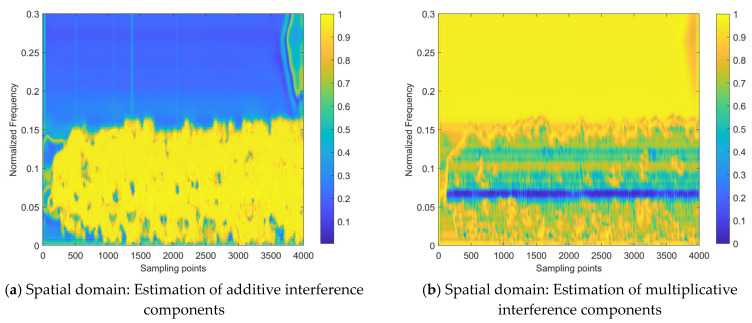

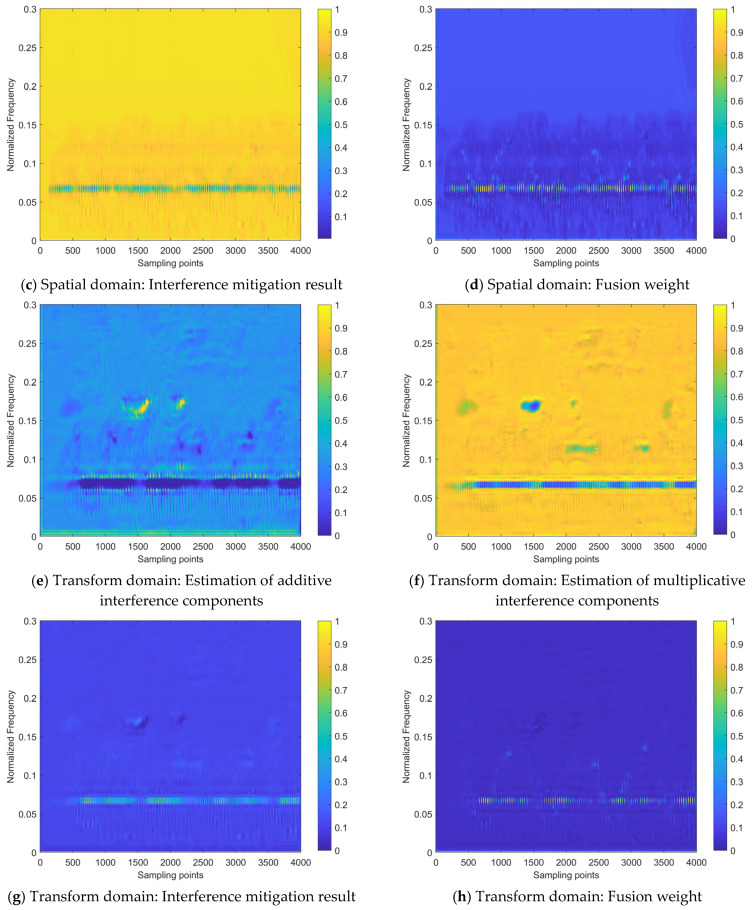
Visualization of interference mitigation process of the dual-domain fusion model on sample #1.

**Figure 14:**
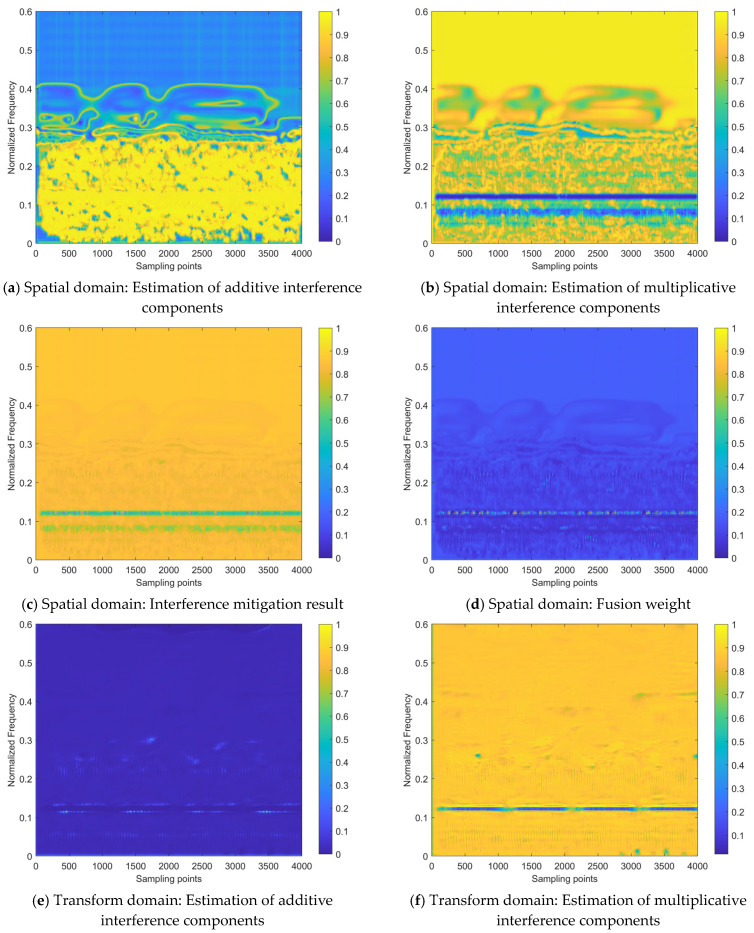

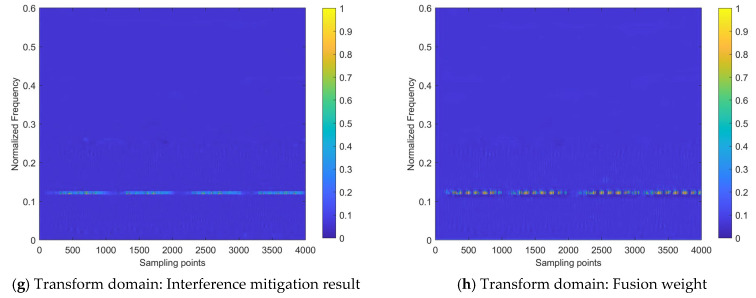
Visualization of interference mitigation process of the dual-domain fusion model on sample #2.

To verify the effectiveness of the proposed network model design, the internal processes for the two samples shown in Figure 12 are visualized, as shown in Figure 13 (corresponding to Sample #1) and Figure 14 (corresponding to Sample #2). All sub-figures of Figure 13 and Figure 14 are presented in the heatmap format, after normalizing the feature maps in the neural network to a range between 0 and 1, where the lower the element value in the feature map, the lower the activation intensity in the heatmap, indicating a suppressed state; the higher the value, the higher the activation intensity, indicating an activated state.

From the interference mitigation approach based on the a priori setting proposed, as shown in Equation (15), it can be concluded that the estimate of the additive interference component is utilized to subtract from the strength of the interfered time–frequency distribution image for cancellation, while the estimate of the multiplicative interference component serves as the denominator (ranging from 0 to 1), to achieve intensity multiplication. For the estimates of additive or multiplicative interference components in Figure 13 and Figure 14, the higher the activation intensity in a region, the closer its value is to 1, and the lower the activation intensity, the closer its value is to 0. Thus, for the input time–frequency distribution image, subtracting the regions with high activation intensity in the feature map of the additive interference component estimate helps to mitigate the interference components. Similarly, dividing by the regions with low activation intensity in the feature map of the multiplicative interference component estimate helps to enhance the strength of the target echo signal components.

Figure 13 and Figure 14 demonstrate that the target echo signal’s regional activation intensity is notably lower when compared to other affected regions within the transform domain-based estimate of both additive and multiplicative interference components. This suggests that the processing based on the transform domain is more inclined to recover the target echo signal component by directly addressing the estimate of the multiplicative interference component. On the other hand, spatial domain-based processing involves attributing most of the interference components to additive interference components and achieving interference cancellation. Subsequently, the recovery of the target echo signal component is realized by dividing the area where the target echo signal component is located by a smaller number. On the other hand, the approach based on the spatial domain attributes the majority of interference components to additive interference and achieves interference cancellation. It then proceeds to recover the target echo signal component by dividing the region where this component is present by a smaller numerical value.

Regarding the heat maps of interference mitigation results and fusion weights, the regions with high activation intensity represent the recovery results of the target echo signal component. The final interference mitigation result is obtained by multiplying the interference mitigation result of a single feature domain by the corresponding fusion weight, then summing the dual-domain results, as shown in Figure 10. Two related phenomena can be found in Figure 13 and Figure 14:

(1) Among the interference mitigation results of the two feature domains, the results from the transform domain are closer to the final interference mitigation results shown in Figure 12 than those from the spatial domain;
(2) The analysis of Figure 12 mentioned that there is an energy residual band with irregular distribution along the time axis and frequency lower than that of the target echo signal component in the final interference suppression results for Sample #2. This is due to the inaccurate estimate of the multiplicative interference components in the spatial domain, as shown in Figure 14. It can be observed that there are two prominent regions with low activation intensity in Figure 14b,c, where the region with lower position and lower continuity is the error result introduced by the spatial domain-based processing.

These two phenomena demonstrate the effectiveness of the introduced transform domain-based processing approach and its superiority over the spatial domain-based processing approach.

Although the interference mitigation results in the spatial domain are not ideal, the fusion weights limit the contribution of these results to the final interference mitigation results, thereby preventing the further propagation of error results. Specifically, for the interference mitigation result of the single domain, only the area with high activation intensity in the fusion weight is allowed to provide a reference with a higher weight for the determination of the final interference mitigation results.

Therefore, the inaccurate areas in the spatial domain interference mitigation results do not significantly affect the final results. It can be observed that the role of the fusion weight is similar to the element-level control bit of the matrix. Of course, this does not mean that the fusion weight is more important than the interference mitigation result. The importance of both should be equal and they should be viewed as mutually reinforcing. This is because the fusion weights in the dual-domain fusion module are not defined independently, but are further evolved based on the interference mitigation results of that feature domain, as shown in Figure 10. Without the ability to extract complex features based on a certain domain, provided by the interference estimation network, it would be impossible to obtain the interference mitigation effect illustrated in Figure 12 by relying on fusion weights alone.

### 5.2. Comparative Experiment

#### 5.2.1. Comparison with Other Time–Frequency Filtering Methods

This section provides the experiment with the comparisons among the proposed dual-domain fusion model and other end-to-end CNN models, which aim to validate the superior performance and efficiency brought by the design of the proposed model. Since there are rare neural network-based solutions that match the task addressed in this paper, the models selected for comparison were primarily designed for image denoising. The principles of image denoising are closely related to the time–frequency image interference mitigation task, and both are categorized as end-to-end image restoration tasks. Therefore, the models for comparison can be easily applied to the concerned task via transfer learning.

**Figure 15:**
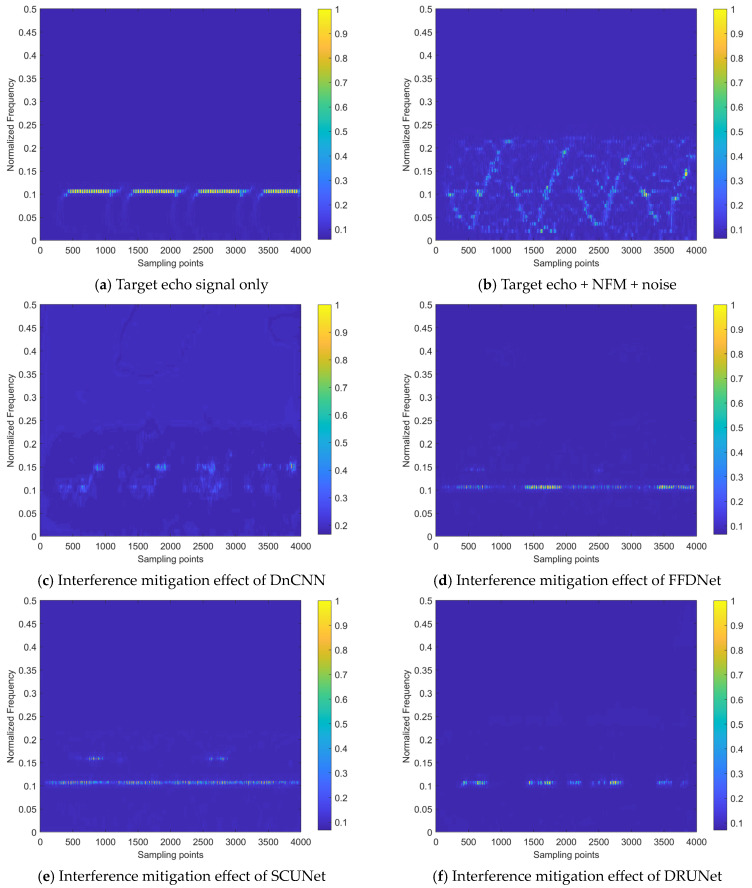

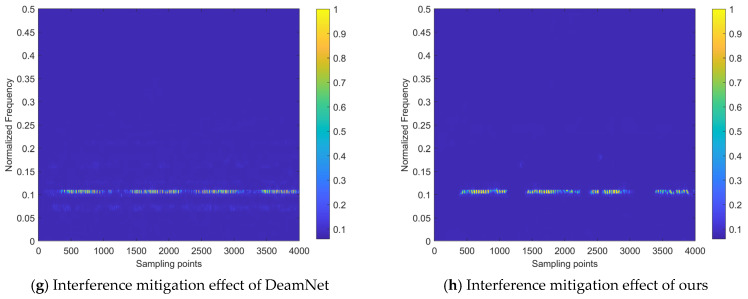
Comparison of NFM interference mitigation effects of various neural network models.

The models used for comparisons include DnCNN [48], FFDNet [49], SCUNet [50], DeamNet [51], and DRUNet [52]. To ensure fairness, they are applied to the same training configuration. The comparison experiments are conducted under the SNR of 0 dB, taking the real-valued time–frequency distribution coefficients interference mitigation task of an NFM signal with the ISR of 5 dB as an example. The results are shown in Figure 15.

It can be observed from Figure 15 that after the superimposition of interference and noise, the intensity of the target echo component is significantly suppressed, making it difficult to obtain the target information. The DnCNN model removes some of the energy distribution caused by noise and interference, but still leaves residual energy around the frequency where the target echo signal component is located. It has the weakest performance of all the models. FFDNet, SCUNet, and DRUNet have better interference mitigation effects, compared to DnCNN, but still lack the ability to recover the periodic information of the beat signal. Additionally, the results of FFDNet and SCUNet still have residual interference energy at other frequencies. The interference mitigation effect and periodicity recovery effect of DeamNet on the beat signal show certain improvements compared to the aforementioned models, but there is still a faint residual energy. Our dual-domain fusion network model has the best mitigation effect on interference components and the best comprehensive recovery effect on the target echo signal, which proves the effectiveness of its design and the performance advantages it brings.

Table 3 provides the overall performance of the compared models on the validation set.

In Table 3, for the evaluation of the time–frequency image restoration task, the PSNR value of our model reaches 32.652 dB, with its SSIM value reaching 0.976, indicating superior restoration performance. On the other hand, the model with the most limited restoration capability is the DnCNN, which is far below the other models, which is basically consistent with the relative trend shown in Figure 15.

In terms of the time–frequency energy aggregation degree after interference mitigation, the Rényi entropy values of the majority models, except for DnCNN, show relatively small differences. This metric is greatly influenced by the interference suppression effect, but less affected by the recovery effect on the target echo signal. In other words, the interference mitigation results achieved by these models (excluding DnCNN) have a similar time–frequency energy aggregation degree, which is consistent with the effects shown in Figure 15.

In terms of signal quality, our model has the highest ∆SINR metric value, indicating that the residual interference and noise energy in the interference mitigation results achieved by the proposed interference mitigation method are the lowest. Therefore, compared to other models, it is able to achieve the energy characteristics of the target echo signal closer to the interference-free state.

A quantitative analysis of the aforementioned models was conducted using the computational complexity evaluation metrics, with the results presented in Table 4. It should be noted that there is no clear linear relationship between FLOPs and params due to the significant differences in the structural design and operational principles of the various models. In other words, a larger param does not indicate a larger FLOPs, the latter is also closely related to the manner in which feature maps are processed.

The proposed dual-domain fusion model mainly consists of two improved UNet++ network modules for the interference estimations of the spatial domain and transform domain, respectively. Moreover, each domain estimation module has the interference estimator branch, implemented by the cascaded dimensional attention module, serving outputting additive and multiplicative interference estimates. The DTCWT with inverse transform is used for the transform domain part. Thanks to the lightweight designs of the RGC module and the bottleneck module, as well as the restriction on the feature map channel number at each level of the UNet++ module, the entire model has significantly lower params and FLOPs compared to other models.

#### 5.2.2. Mitigation Effect on Different Interference Types

**Figure 16:**
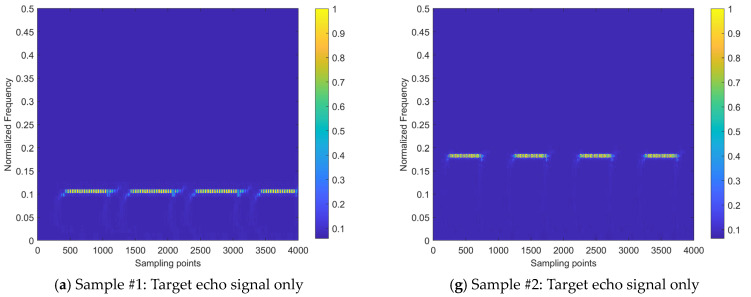

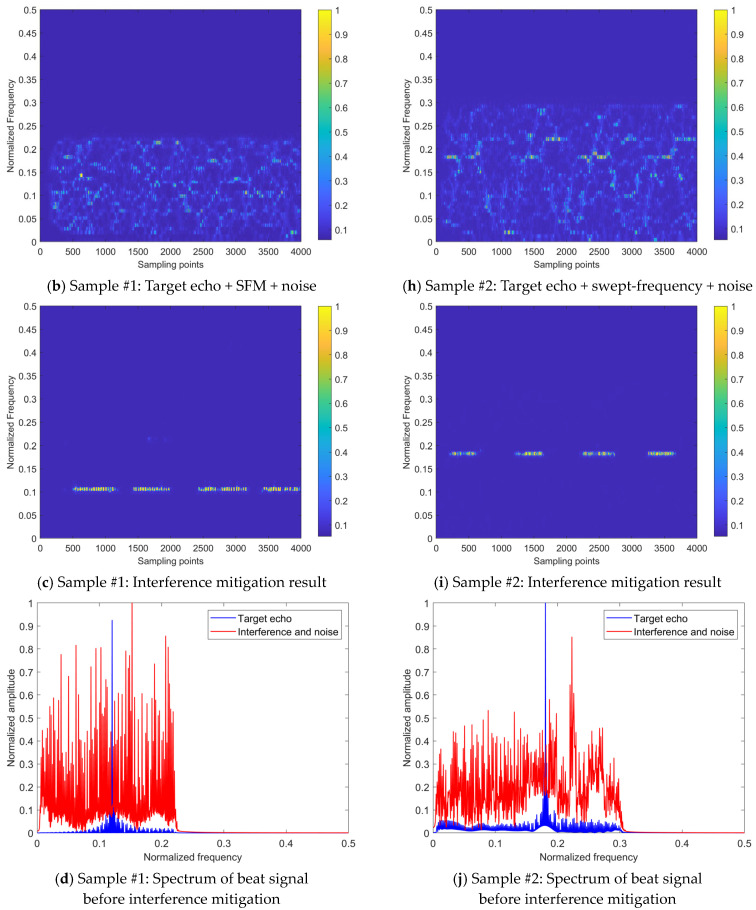

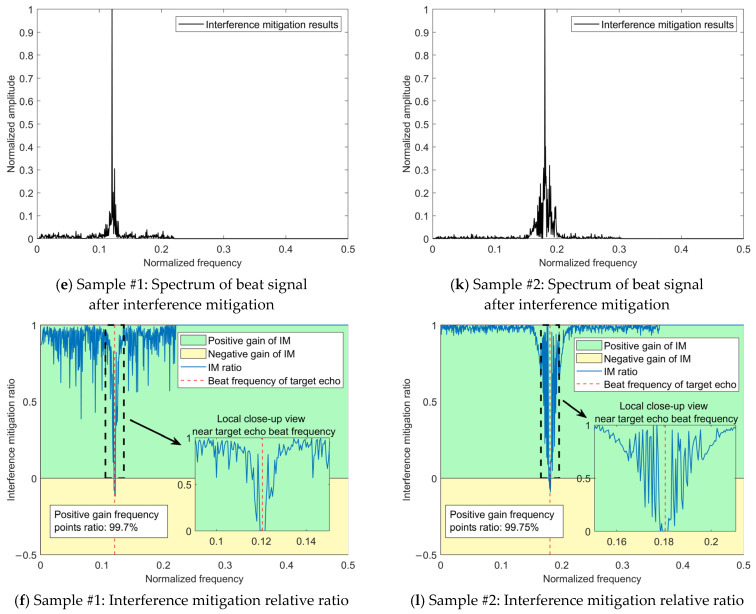
Mitigation effects of the proposed method on SFM and swept-frequency interference signals.

Figure 16 demonstrates the effects of the proposed method in mitigating the SFM interference signal and swept-frequency interference signal, with an ISR of 10 dB. Table 5 presents the overall performance of the mitigation of the proposed method on these two types of interference signals on the validation set.

Figure 16 illustrates that the proposed method removed the most energy from two kinds of interference signal and restored the beat frequency signal components of the target echo at the correct frequency. On the other hand, there is still a certain degree of error in the restorations of the target echo signal energy distribution, based on the time axis.

From Figure 16 and Table 5, it can be seen that the proposed method, based on the task form of time–frequency distribution image restoration, has good interference mitigation performance on various types of non-coherent interference signals. Thus, it can help the FMCW radar to avoid interference and operate normally.

## 6. Discussion

### 6.1. About the Difference between Spatial Domain and Transform Domain

As the results show in Section 5.1, the interference mitigation effect achieved by implementing the image transform domain was superior to that based on the traditional spatial domain. We tried to find the reasons for this from the perspective of the principles and characteristics of the transform implementation of DTCWT.

The processing based on the spatial domain refers to operations directly performed on the image. Its advantage lies in the fact that no transform of the image is required, thus the computational process is simple and straightforward. However, this operation may not be able to fully capture the high-frequency details and texture information, especially when the interference and noise in the time–frequency image are mixed with the echo signal components.

DTCWT provides multi-scale and multi-directional feature information of the time–frequency distribution image. After implementing the domain transform of DTCWT, the energy distribution could be decomposed into different frequency sub-bands, each of which contains unique information such as image texture, edges, contours, etc. This is important for interference mitigation, as various interference and noise components usually manifest as irregular shapes within the image, and most of them could be separated via DTCWT. In our model, they are further enhanced in the estimator branches through the mechanism of dimensional attention, making it easier to estimate the interference.

### 6.2. About the Essence of Interference Mitigation on Time–Frequency Distribution

For the time–frequency distribution of the received signal, noise and interference affect its state of energy distribution; assuming the radar received signal contains only the target echo, and its time–frequency energy distribution state is denoted as state a. Upon the influence of noise, state a evolves into state b. Further, when state b is subjected to different types of interference, it evolves into states $c_{1},c_{2},c_{3}$, etc. That is, these factors cause the time–frequency energy distribution of the received signal to evolve from state a to any arbitrary state x. Due to the non-stationary of these factors, state x is actually dynamically varying within a certain range. As shown in Figure 4, the interference estimates provided by the neural network are essentially approximate quantitative representations of the energy distribution’s state transition process, that is, an increment state δ with entropy-increasing characteristics from state a to state x.

Based on the above perspective, the cancellation of the increment state δ, achieved through the additive and multiplicative interference component, can essentially be regarded as an extended version of the least squares model to a broader context. Therefore, the smaller the range of this dynamic change, the more conducive it is for the neural network to accurately learn this process; conversely, the greater the intensity of noise and interference factors, the larger the range of dynamic change, and the weaker the neural network’s ability to master this process. When the above relationship is applied to the signal’s time–frequency distribution image, the factors affecting the dynamic change range are the type and intensity of interference.

## 7. Conclusions

This paper proposed an interference mitigation method based on time–frequency analysis and convolutional neural networks for non-coherent interference on FMCW radar. It makes an a priori assumption on the interference signal characteristics, including additive and multiplicative interference components, and proposes a model for interference mitigation based on interference estimation and cancellation. The method achieved interference mitigation in both the spatial domain and the transform domain of the radar beat signal’s time–frequency distribution image. Furthermore, the dual-domain fusion approach yielded a superior interference mitigation effect. The transform domain approach was implemented by a two-dimensional dual-tree complex wavelet transform, and the dual-domain interference estimates were achieved through a structurally improved convolutional neural network model of UNet++.

The proposed interference mitigation method was evaluated from three perspectives; the restoration effect of the time–frequency image, the time–frequency energy aggregation degree, and the signal quality after interference mitigation. Simulation experiments proved the rationality and effectiveness of the model design and verified its good mitigation capability for different types of non-coherent interference signals, which is validated to have superior performance over other similar CNN model-based approaches.

## Figures and Tables

**Table 1:** Parameter configurations of simulation.

Object	Parameter	Value
Radar signal	Center frequency	4 GHz
	Bandwidth	50 MHz
	Pulse width $T_{p}$	10 μs
	Chirp rate	5 MHz/μs
	Sampling frequency	100 MHz
Target echo	Range *	50~1000 m
Background	Signal-to-noise ratio (SNR)	0 dB
Interference signal	Interference-to-signal ratio (ISR) *	0~10 dB
	Phase *	0~2π
	Interference and jamming signal type	NFM, SFM, swept-frequency
	Delay time *	−5~5 μs
	Bandwidth *	50~80 MHz
	Period of swept-frequency *	5~50 μs
	Pulse width of SFM *	5~50 μs
Time–frequency analysis	Implementation method	Fourier synchrosqueezed transform (FSST)
	Window type	Kaiser window
	Window length	256
	Window shape factor β	10
	Overlap length	255
	FFT points	256

* The value is randomly sampled from the uniform distribution with the provided range.

**Table 2:** Comparison of the average interference mitigation performance of the three models on the validation set samples.

Task Type	Evaluation Metric	Spatial Domain Model	Transform Domain Model	Dual-Domain Fusion Model
Image restoration	PSNR (dB)	30.385	31.452	32.652
	SSIM	0.964	0.971	0.976
Time–frequency energy aggregation degree	RE	15.9959	15.9895	15.9879
Signal quality	∆SINR (dB)	14.4838	16.3621	18.8837

**Table 3:** The average mitigation performance of various neural network models on NFM interference on validation set samples.

Task Type	Evaluation Metrics	DnCNN	FFDNet	SCUNet	DRUNet	DeamNet	Our Model
Image restoration	PSNR	29.162	31.783	31.721	32.149	32.543	32.652
	SSIM	0.859	0.943	0.959	0.961	0.972	0.976
Time–frequency energy aggregation degree	RE	16.3693	16.1164	16.0467	15.9982	15.9793	15.9879
Signal quality	∆SINR (dB)	10.5142	14.9836	16.5562	17.7846	18.2215	18.8837

**Table 4:** Computational complexity comparison of various models.

Metrics	DnCNN	FFDNet	SCUNet	DRUNet	DeamNet	Our model
Params	1.2 × 10^6^	1.1 × 10^6^	32.6 × 10^6^	17.9 × 10^6^	1.8 × 10^6^	0.8 × 10^6^
FLOPs	309.8 × 10^9^	68.5 × 10^9^	554.5 × 10^9^	319.1 × 10^9^	582.8 × 10^9^	11.2 × 10^9^

**Table 5:** The average mitigation performance of the proposed method on the interference signals of SFM and swept-frequency on validation set samples.

Task Type	Evaluation Metrics	SFM	Swept-Frequency
Image restoration	PSNR	31.673	32.421
	SSIM	0.977	0.984
Time–frequency energy aggregation degree	RE	15.9473	15.8966
Signal quality	∆SINR (dB)	16.3425	18.0752

## Data Availability

The data are available from the corresponding author upon reasonable request.
